# Augmented Reality in Medicine: Systematic and Bibliographic Review

**DOI:** 10.2196/10967

**Published:** 2019-04-26

**Authors:** Martin Eckert, Julia S Volmerg, Christoph M Friedrich

**Affiliations:** 1 Department of Computer Science University of Applied Sciences and Arts Dortmund Dortmund Germany; 2 Institute for Medical Informatics, Biometry and Epidemiology University Hospital Essen Essen Germany

**Keywords:** mixed/augmented reality, medicine, mobile computing, systematic review, mobile phone

## Abstract

**Background:**

Augmented reality (AR) is a technology that integrates digital information into the user’s real-world environment. It offers a new approach for treatments and education in medicine. AR aids in surgery planning and patient treatment and helps explain complex medical situations to patients and their relatives.

**Objective:**

This systematic and bibliographic review offers an overview of the development of apps in AR with a medical use case from March 2012 to June 2017. This work can aid as a guide to the literature and categorizes the publications in the field of AR research.

**Methods:**

From March 2012 to June 2017, a total of 1309 publications from PubMed and Scopus databases were manually analyzed and categorized based on a predefined taxonomy. Of the total, 340 duplicates were removed and 631 publications were excluded due to incorrect classification or unavailable technical data. The remaining 338 publications were original research studies on AR. An assessment of the maturity of the projects was conducted on these publications by using the technology readiness level. To provide a comprehensive process of inclusion and exclusion, the authors adopted the Preferred Reporting Items for Systematic Reviews and Meta-Analyses statement.

**Results:**

The results showed an increasing trend in the number of publications on AR in medicine. There were no relevant clinical trials on the effect of AR in medicine. Domains that used display technologies seemed to be researched more than other medical fields. The technology readiness level showed that AR technology is following a rough bell curve from levels 4 to 7. Current AR technology is more often applied to treatment scenarios than training scenarios.

**Conclusions:**

This work discusses the applicability and future development of augmented- and mixed-reality technologies such as wearable computers and AR devices. It offers an overview of current technology and a base for researchers interested in developing AR apps in medicine. The field of AR is well researched, and there is a positive trend in its application, but its use is still in the early stages in the field of medicine and it is not widely adopted in clinical practice. Clinical studies proving the effectiveness of applied AR technologies are still lacking.

## Introduction

### Background

Augmented reality (AR) is a technology that extends the user’s reality using digital information. It has become a publicly discussed topic in our society and a prime field for new kinds of apps in the medical sector. AR can be seen in many aspects of medicine; for example, Kamphuis et al reported that AR technologies have started maturing in the field of anatomical and physiological education [1]. There is a high demand for assisting systems due to increased stress in public health systems, which is one of the reasons for the fast development within the field of AR and virtual reality (VR).

The focus of this systematic and bibliographic review is to provide insight into the research conducted in the field of AR. The scope lies in its advances in the medical field, centering on the medical specialty, technical impact, maturity of projects, and publications on the topic of AR in medicine.

Recently Chen et al [2] published a review covering the development of AR technology in the medical field. Their study thematically overlaps with this review paper, but in their paper [2], only Scopus [3] was used as a data source and a text mining approach was used to analyze the retrieved data. In contrast, this review uses manual checking, broader categorization, and the PubMed database in addition to Scopus.

Within the review presented here, the publications have been manually categorized and structured according to medical branches, applied technologies in hardware and software, and strong indicators of the maturity of the developed technology.

For the classification of AR apps, the taxonomy proposed in the Handbook of Augmented Reality by Hugues et al [4] was used. The taxonomies by Schmalstieg et al [5] and Aukstakalnis [6] were used to categorize AR displays and the technique of tracking. For the assessment of maturity, the technology readiness level [7] was used, which is a method to rank and analyze the demonstrated technologies.

The aim of this review paper was to build a foundation and guide to the literature and to be used as a motivation for scientists, researchers, and developers in the field of AR in medical settings. The main objectives were to assess the current state of research, identify possible future trends, and provide an overview. Another outcome of this research was an interactive table (Multimedia Appendix 1) of the research conducted in AR in the field of medicine from March 2012 to June 2017.

### Overview of Augmented Reality Technology

This section offers a brief overview of AR and its definitions, differentiating it from VR. Additionally, a short overview of its technical development and its applications will be given.

There are several definitions of AR, depending on how it enhances our environment with artificially added information and whether one can interact with this information [8].

AR is a variation of VR [9]. In contrast to VR, a user of an AR system always experiences their own reality in real-time. A VR system always has a synthetic feature, and it imitates reality rather “than supplements the real world” [10], although there are VR systems that imitate AR by using cameras showing the user his/her surroundings augmented with additional information. Azuma et al [9] reported that a VR environment is a completely synthetic environment that separates the user from reality.

**Figure 1:**
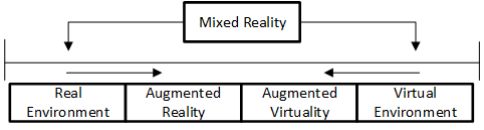
“Reality-Virtuality Continuum” by Milgram et al [11].

Another term used in this context is mixed reality (MR), which could be explained by the “reality-virtuality continuum” explained by Milgram et al [11]. MR shows the reality at one end and VR at the other end, with AR and augmented virtuality (AV) lying between the two ends (Figure 1).

Milgram et al used the term MR to distinguish different MR displays and design a taxonomy for categorization of MR systems [11]. In addition, augmentation through senses other than vision is important, but not as common. The auditory sense or haptic sense is an additional source of information. Each origin of additional information can be classified as AR/MR.

From a historical viewpoint, the first approach to AR was the Sensorama, a machine that was supposed to provide a cinematic experience with all senses [8]. It was developed by Heilig in the 1950s and was the first documented reference to AR, although at that time, there was no distinction between AR and VR. In 1968, Sutherland [12] developed a head-mounted display (HMD), which made it possible to experience AR and VR environments for the first time. The first reference to AR as a term was made by Caudell, a researcher at Boeing who coined the term in 1990 [13]. Two years later, Caudell and Mizell [14] developed an early prototype, which enabled technicians to project blueprints onto a surface.

With the invention of handheld devices like smartphones and tablets, there were opportunities to advance to a bigger audience. In 2013, Google presented Google Glass, an HMD that provides hands-free interaction via a voice interface and enables the user to call, send texts, or search the internet. In 2015, Microsoft presented the HoloLens; this device allows one to see and interact with holographic 3D virtual objects via voice, gaze, and gestures.

## Methods

This section provides an overview of the methods used to acquire relevant publications in addition to the restrictions and inclusion and exclusion criteria used. Thereafter, an introduction to the principles used to analyze the publications and the relevant variables are presented.

### Acquisition of Publications

To gather the initial data, a query was designed to collect publications from the PubMed [15] and Scopus [3] databases, containing the keywords “augmented” and “reality” restricted to the period between May 15, 2012, and June 30, 2017. The search for Scopus was additionally restricted by the subject area medicine,” and the timeframe was manually readjusted to the timeframe used for PubMed. The term “mixed reality” was applied synonymously to AR, but was not explicitly searched for.

In three stages, the results of the query were filtered using a set of factors to determine the validity and relevance for this review. After each exclusion stage, the remaining papers were analyzed, and the corresponding variables were entered into the results file. We then processed each batch individually. To ensure reliability during the classification phase, a subset of 85 (25%) of 338 publications were cross-checked by the authors, and conflicts were resolved by consensus.

In the next step, data were analyzed and prepared for visualization, where applicable. Analysis and visualization were conducted using R (version 3.4.2; “Short Summer”) [16].

### Data Analysis

#### Exclusion and Inclusion Criteria

**Figure 2:**
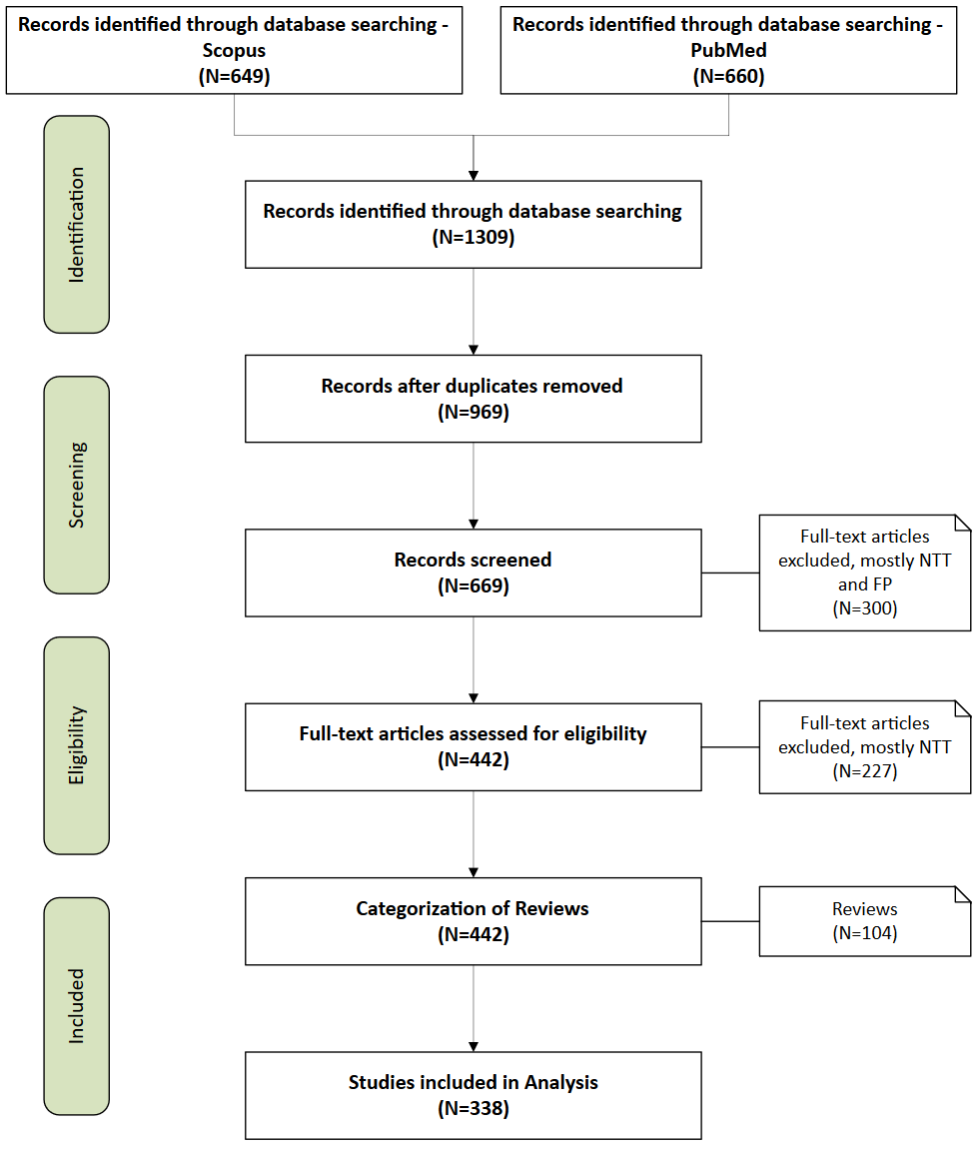
Filtering Process of the initial query and remaining results. NTT = No Treatment or Training, FP = False Positive.

The initial queries returned 1309 results (Multimedia Appendix 2), from which 340 duplicates were removed, leaving 969 eligible publications. The authors of these publications were contacted if the publications were not accessible. Subsequently, mismatched publications were excluded in three iterations, yielding a total of 338 publications. The last iteration was an additional categorization of review publications (Multimedia Appendix 2). A visual overview of the filtering process is shown in Figure 2, which follows the Preferred Reporting Items for Systematic Reviews and Meta-Analyses flowchart [17]. The complete dataset analyzed, along with the results in the form of an interactive table, is available in Multimedia Appendix 1. For a complete reference list, see Multimedia Appendix 3.

Table 1 shows the exclusion criteria. The main reason for exclusion of a publication was the absence of a concrete connection to medical treatment or training of health professionals, or “No Treatment or Training,” such as papers about the influence of Pokémon Go on college students [18].

The second main reason for exclusion was a false positive result, a criterion that includes publications without any connection to AR because they either focus entirely on VR or separately contain the tags “augmented” and “reality.” This included the paper by Yoo et al about the effect of training alone or training with VR, both augmented by electromyography, for children with cerebral palsy [19].

The exclusion criteria “Other” refers to papers where only the abstract could be found, papers where the authors did not reply, conference posters, collections of papers, and books. One additional criterion for exclusion was veterinary publications, for example, the paper by Sutton et al about the glass knife-fish and the way it uses “electrosensory feedback” to hold a position in a moving environment [20].

**Table 1:** Overview of exclusion criteria and number of excluded publications.

Exclusion criteria	Number of excluded publications
No treatment or training	257
False positive	173
Paper not in English	13
Veterinary medicine	3
Other	81
Duplicates	340
Review	104
Total	971

**Table 2:** Overview of the investigated variables and the related classification method.

Variable	Classification method
Year of publication	Metadata
Geolocation	Metadata
MeSH^a^ data	MeSH [21]
Medical scope	Manual
Interactive or haptic	Manual
Collaboration	Manual, binary
Clinical trial	Clinicaltrials.gov [22] and manual, binary
Augmented reality taxonomy	Hugues et al [4] and manual
Technology readiness level	US Department of Defense [7] and manual
Display	Schmalstieg et al [5] and manual
Tracking	Schmalstieg et al [5] and manual

^a^MeSH: Medical Subject Headings.

#### Summary of Variables

A short overview of the variables used is presented in Table 2.

#### Classifications

To classify the publications, several factors were chosen to create a comparable dataset within the research of AR applied to medicine. Besides the classification methods from the metadata, such as the year of publication and geographical location of the first author, several other classification factors were manually introduced into the dataset.

##### Medical Subject Headings

For medical classification, the Medical Subject Headings (MeSH) terms were used. These terms, only applicable to the PubMed database, offer a terminology for categorization of biomedical apps and can be used, for example, to categorize publications in PubMed. For this review, the 2017 version of MeSH terms was used.

##### Clinical Trial

For additional classification, data from ClinicalTrials.gov [22] were used. The database assesses if the publication, in case it is a prototype or product, was tested in a clinical environment during a study and if it was registered on ClinicalTrials.gov.

##### Collaboration

This variable was used to assess how collaboration using the AR device was realized. This could be done via a remote connection. For example, this category included studies in which two medical professionals present at different locations see the same AR environment and are allowed to interact with it.

##### Research Maturity

The technology readiness level assessment is a method to estimate the maturity of technology (Table 3). Usually, the level ranges from 1 to 9, where 1 is the least and 9 is the most matured technology. It was introduced by the US Department of Defense to rate technologies. The US Department of Defense definition from 2011 [7] was used to determine and quantify the state of research projects in this paper.

**Table 3:** Technology readiness levels according to the US Department of Defense [7].

Technology readiness level	Description
1	Basic principles observed and reported
2	Technology concept or application formulated
3	Analytical and experimental critical function or characteristic proof of concept
4	Component or breadboard validation in a laboratory environment
5	Component or breadboard validation in a relevant environment
6	System/subsystem model or prototype demonstration in a relevant environment
7	System prototype demonstration in an operational environment
8	Actual system completed and qualified through test and demonstration
9	Actual system proved through successful mission operations

**Figure 3:**
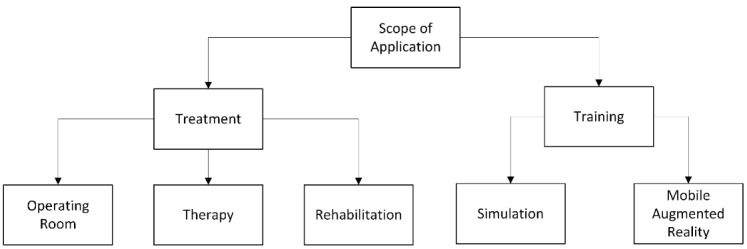
Medical scope criteria for application.

##### Medical Scope

The scope of the published apps was manually split into two subgroups—treatment and training—as shown in Figure 3. The treatment group was further subdivided into three groups: operating room, therapy, and rehabilitation. The training group was subdivided into simulation and mobile AR. The subgroups originated after the short initial overview of the publications.

##### Augmented Reality Display

In this section, we present a short introduction to the AR display technology. Schmalstieg et al [5] divided the placement of the display technologies into three spaces: “head,” “body,” and “world.” The placement can be identified by looking at where the display is stationed. “Desktop displays, Virtual Mirrors, Virtual Showcase, Window and Portal Displays and Projector-based displays” [5] are located in a fixed place in the world; “Handheld displays” [5] are stationed on the body; and a “Near-Eye Display” [5] is placed on the head of the user.

###### Head Location

The HMD is the most well-known display technology used for AR. These displays are further classified into “Optical-See-Through Head-Mounted Display” and “Video-See-Through Head-Mounted Display” [5]. The Optical-See-Through enables a user to see the real world augmented with information via see-through lenses such as the Microsoft HoloLens. Videos see-through displays use an additional camera to provide the user with surrounding reality. Thus, it is not seen directly by the user (eg, HTC Vive). An example of this technology in a medical context is the digital microscope in an operating room.

###### Body Location

The commonly known smartphone and tablet are best examples of handheld AR devices and are mostly known for games like Pokémon Go. The back camera of such devices is used to provide the user with a video see-through image. In a medical context, this could be an app to visualize anatomical structures in a book.

###### World Location

A display located in the world has many advantages for AR. These displays can be divided into desktop display, virtual mirror, projector-based display, and stationary display.

A desktop display can be used as an AR display by adding a webcam, which then provides the necessary input of reality. The display then shows both reality and additional information within it. A virtual mirror uses the front camera of the device and shows a picture of what is in front of the camera. Another kind of an augmented mirror is the visual showcase, but it does not show the user; it is a stationary variant of the optical see-through, which allows the user to see through alongside additional information. The last stationary displays are projector-based displays. They can be dependent or independent from the view.

As another option, one can simply mount the projectors onto an HMD, creating a personal projection with retro-reflection screens. The location “World” can be found in any medical context; a known example here would be in the operating room, where images acquired prior to surgeries are either shown on a display or projected directly onto the patient.

##### Augmented Reality Tracking

Tracking in an AR environment can be categorized by the different technologies used to track objects. In this paper, the applied categorization refers to whether the sensors are stationary or mobile and which type of optical tracking was used [5].

Today, many systems use more than one tracking mechanism; the HTC Vive system uses stationary tracking in the form of stationary lighthouses in the room with beam detectors in the tracked devices and mobile tracking in the form of gyro sensors.

###### Stationary Tracking Systems

Stationary tracking is a technique that uses either a mechanical device or an electromagnetic field, infrared light, or ultrasound to obtain position data. Commonly known stationary tracking systems are the SteamVR trackers, which use a time-based location estimation for the HTC Vive or other AR/VR solutions. Another option is Ultrasonic Tracking [5], which uses an ultrasonic pulse as a time-of-flight source. Therefore, it is possible to track a position by measuring the time a pulse needs from the source to the sensor.

###### Mobile Tracking Systems

While stationary tracking does not allow the user to move around much, mobile sensors allow tracking outdoors. The most popular mobile sensor is the Global Positioning System, which determines the position of an object by the time of flight of signals emitted by a satellite. Inside an already established wireless network, it is also possible to track an object simply by the base station used to connect to the wireless network.

###### Optical Tracking

There are two ways to classify tracking in AR: “model-based versus model-free tracking” and “markers/fiducial versus natural features” [5]. Model-based tracking uses an existing model that is created beforehand. The model-free tracking is an on-the-fly technique, with a temporary model. This allows for more flexibility, especially if there is a combination of 3D tracking and 3D scanning. Marker tracking, also known as fiducial tracking, offers a possibility for more robust algorithms because markers are previously known patterns that are more easily recognized. Natural feature markers often require higher image quality to detect the object.

Interest points, also known as key points, are mostly used to track an object, but the key points should be easily detected and stable from all angles. An alternative for interest points is edge features, but here, it is necessary to distinguish the edge from the background.

##### Augmented Reality Taxonomy

**Figure 4:**
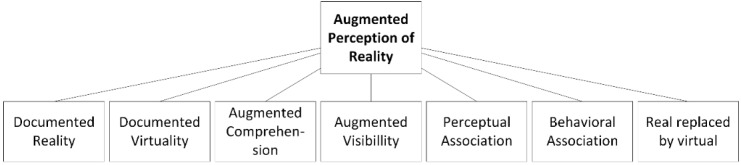
Functional taxonomy according to Hugues et al [4].

In this section, the parts of the “Functional Taxonomy” [4], introduced by Hugues et al, that are used for the classification will be explained. The functionality “Artificial Environment” was excluded, since it defines AR in the context of time. An overview of the part of the taxonomy used is shown in Figure 4.

Six subfunctionalities of augmented perception can be separated by considering the ability to assist in decision making for AR. The first is functionality 0, where real images and virtual entities are shown on the screen but have no relation. This conditioning indicates that there is one screen but two different boxes: One box shows real images and one shows a virtual entity.

###### Subfunctionality 1: Documented Reality and Documented Virtuality

The functionality “Documented Reality and Documented Virtuality” [4] is a minimal function of AR. Augmentation only consists of two boxes: One displays the real images and one displays the virtual entity. However, on distinguishing functionality 1 from functionality 0, real images and virtual entities are related to each other in functionality 1 and therefore provide additional information within the context of reality.

###### Subfunctionality 2: Reality With Augmented Perception or Understanding

In “Reality with Augmented Perception or Understanding” [4] environments, there is only one box left, which is shared by real images and virtual entities. The subfunctionality can be categorized into two levels—“Augmented Understanding” and “Augmented Visibility.” “Augmented Understanding” means that the virtual entities show alignment with real images but are not always close to each other, and “Augmented Visibility” means that the virtual entities cover the real images completely.

###### Subfunctionality 3: Perceptual Association of the Real and Virtual Images

The subfunctionality “Perceptual Association of the Real and Virtual” [4] is divided further into the levels of “Incrustation” [4] and “Integration” [4] of virtual entities on real images; therefore, this functionality has the ability to differentiate between a projection of a tumor on top of an organ and the 3D image of the tumor onto the whole organ.

###### Subfunctionality 4: Behavioral Association of the Real and Virtual Images

The “Behavioural [sic] Association of the Real and Virtual” [4] is a step further from the “Perceptual Association” functionality, as it adds physical properties to virtual objects, specifically the properties of the real object.

###### Subfunctionality 5: Substituting the Real by the Virtual or Virtualized Reality

“Substituting the Real by the Virtual or Virtualised [sic] reality” [4] is a subfunctionality that allows the real scene to be replaced by an artificial image and vice versa. Therefore, it is possible to change the angle of the view, which makes it possible to see not only reality through a camera but also an artificial image from another point of view.

## Results

### Review Publications

The query results contained 104 review publications. Most of the reviews assessed the use of AR in a surgery setting. The review publications can be found in Multimedia Appendix 2.

Khor et al [23] provided insight into the use of AR and VR, with an emphasis on the surgical workplace. Thomas et al [24] highlighted a computer-aided medicine revolution. Moglia et al [25] provided a review of VR and AR simulators for robot-assisted surgery. Robotic surgery is also the topic of another publication from 2015 [26]. Slade et al provided an assessment [27] of the use of wearable technology in a surgical setting. Bluemel et al [28] gave an overview of freehand single-photon emission computed tomography (SPECT) for navigation and radio-guided surgical procedures like sentinel lymph node biopsies.

These publications do not include neurosurgical reviews. However, Pelargos et al [29] refined a review that covers the historical development, current use, and emerging applications of AR and VR in the field of neurosurgery. A recent study provided a systematic overview of technologies using AR in the field of neurosurgery [30]. Marcus et al [31] published an overview of robotics in keyhole transcranial endoscope-assisted microsurgery.

In total, two publications assessed the use of AR technologies in Urology. One study [32] provided an overview of the history and current state of pediatric robotic surgery, mainly in India. It covered the topic of AR in the outlook, assessing its potential to support pediatric robotic urology. Hamacher et al [33] published a paper on the development of VR, AR, and MR in existing consumer products, in which the main emphasis was on VR. This review paper covers the influence of these technologies used in urology.

Several endoscopic devices are equipped with AR technologies. Mahmud et al [34] provide a prognosis to integrate AR technology into an endoscopic device. They emphasized the importance of collaboration between computer scientists and physicians. Feussner et al [35] stressed upon the importance of a close collaboration between programmers and physicians. They compiled an extensive overview of available technologies and identified associated technological problems. They also estimated the time to bring the technologies to a broadly applicable system.

Smith et al described the use of AR in education in the fields of Obstetrics and Gynecology [36]. There is an emphasis on the change in clinical education, to empower students to use new technologies such as VR and AR devices as well as holograms while teaching in a focused and comprehensive manner.

### Year of Publication

Table 4 shows the distribution of publications over the time. We observed an increase in the number of papers published from 2012 to 2014, a decrease in 2015, and a peak in 2016. The fact that only three papers were electronically pre-published in 2011 can be explained by the rise of electronic pre-publishing in 2011.

### Geolocation

As shown in Table 5, in the majority of publications analyzed, the first author was located in the United States, followed by Germany, Japan, and France. For brevity, this table is cutoff after the top 10 locations.

**Table 4:** Evaluated publications according to publication date from March 2012 to June 2017 (N=338).

Year of publication	Number of publications
2011	3
2012	25
2013	51
2014	81
2015	59
2016	71
2017	48

**Table 5:** Distribution of the top 10 geolocations of the first author (N=264).

Country	Number of publications
United States	73
Germany	40
Japan	27
France	25
China	25
Canada	25
Switzerland	14
Spain	13
United Kingdom	11
Italy	11

**Table 6:** Analysis of the top 10 Medical Subject Headings terms sorted and shown by frequency (N=191).

Medical Subject Headings terms	Number of publications
[Surgery, Computer-Assisted]	77
[Imaging, Three-Dimensional]	65
[Tomography, X-Ray Computed]	39
[Laparoscopy]	24
[Feasibility Studies]	23
[Neurosurgical Procedures]	15
[Pilot Projects]	14
[Computer-Assisted Instruction]	12
[Image Interpretation, Computer-Assisted]	11
[fluoroscopy]	11

**Table 7:** Overview of the results from the variables Clinical Trial and Collaboration (N=338 for each).

Variable	Proposed app tested in a clinical trial	
	Yes	No
Clinical Trial	3	335
Collaboration	16	322

### Medical Subject Headings Terms

The publications from PubMed feature the title and abstract and are annotated with MeSH terms [21]. These publications were not included in the Scopus database. To gain more insight into the nature of the publications, the MeSH terms were analyzed. Of the 267 publications from PubMed, at the time of analysis, only 191 featured MeSH-annotated terms. These terms were added by experts, and therefore, not all recent publications were annotated. The term frequency was calculated, and the results of the top 10 terms are presented in Table 6.

### Clinical Trial

The factor clinical trials was included to analyze if the proposed app was tested in a clinical trial registered in ClinicalTrial.gov [22]. As shown in Table 7, this occurred in three publications, including a study by Ortiz-Catalan [37] who registered their study in ClinicalTrial.gov; this study was about phantom limb pain and aimed to show that treatment with AR decreases pain.

### Collaboration

Table 7 shows the results of a possible collaboration through the app. As shown in most cases, this was not possible; only 16 projects enabled the user to share their AR space simultaneously with other users. This can occur through a remote connection or on a local level, as shown by Vera at al [38] who presented an AR telemonitoring platform to support students learning laparoscopic techniques by overlaying the students’ view with the view of the mentor. Shared open-world displays were not considered to be shareable, even if every computer monitor represents a collaboration.

### Research Maturity

Research maturity, categorized by the technology readiness level index [7], shows a trend in levels 6 and 7 (Table 8). There are also publications that contain research on the effectiveness of AR products; these are represented in level 9. The most common technologies featured in these publications are laparoscopic tools.

### Medical Scope

**Figure 5:**
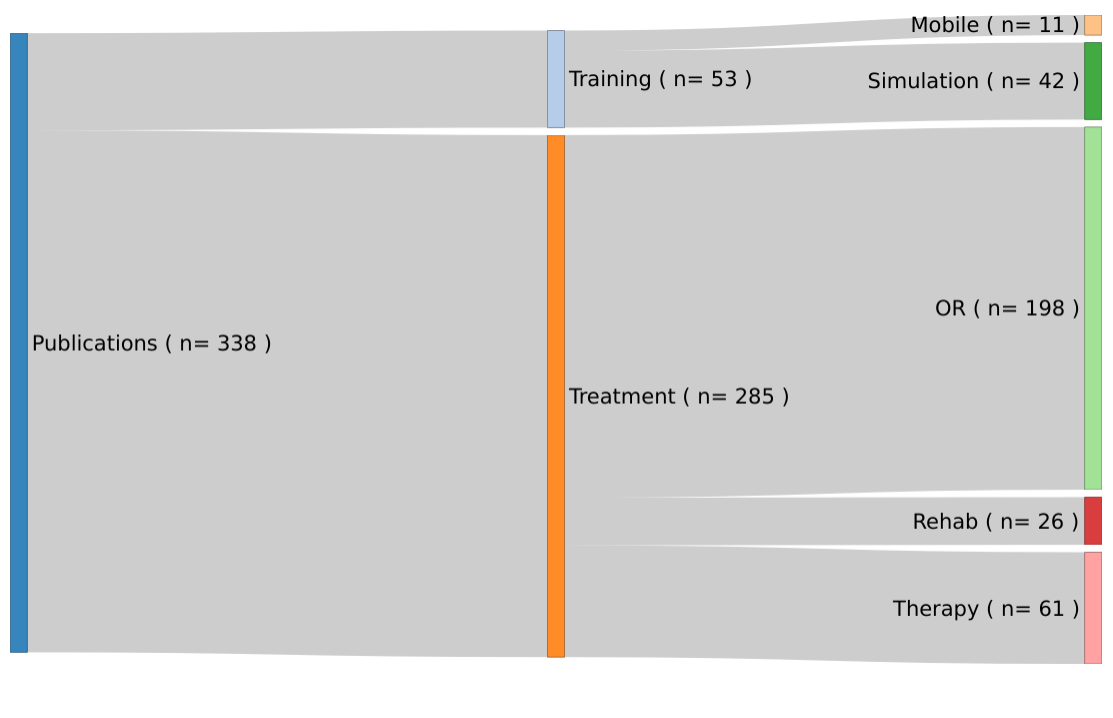
Categorization of medical scope and count of examined augmented reality projects including reviews (N=338).

The results of the Medical scope are presented in Figure 5. Of the 338 publications, 84.3% (n=285) were identified as AR projects that dealt with the actual treatment of patients and 15.7% (n=53) dealt with training scenarios, for example, a clinical simulation feasibility study with Google Glasses [39].

Of the 285 publications identified to be in the treatment category, 69.5% (n=198) projects dealt with scenarios in the operating room and 9.1% (n=26) projects were set in a rehabilitation setting. In addition, 21.4% (n=61) of the presented projects included direct involvement in the therapy of the patient.

### Classification by Schmalstieg et al

**Figure 6:**
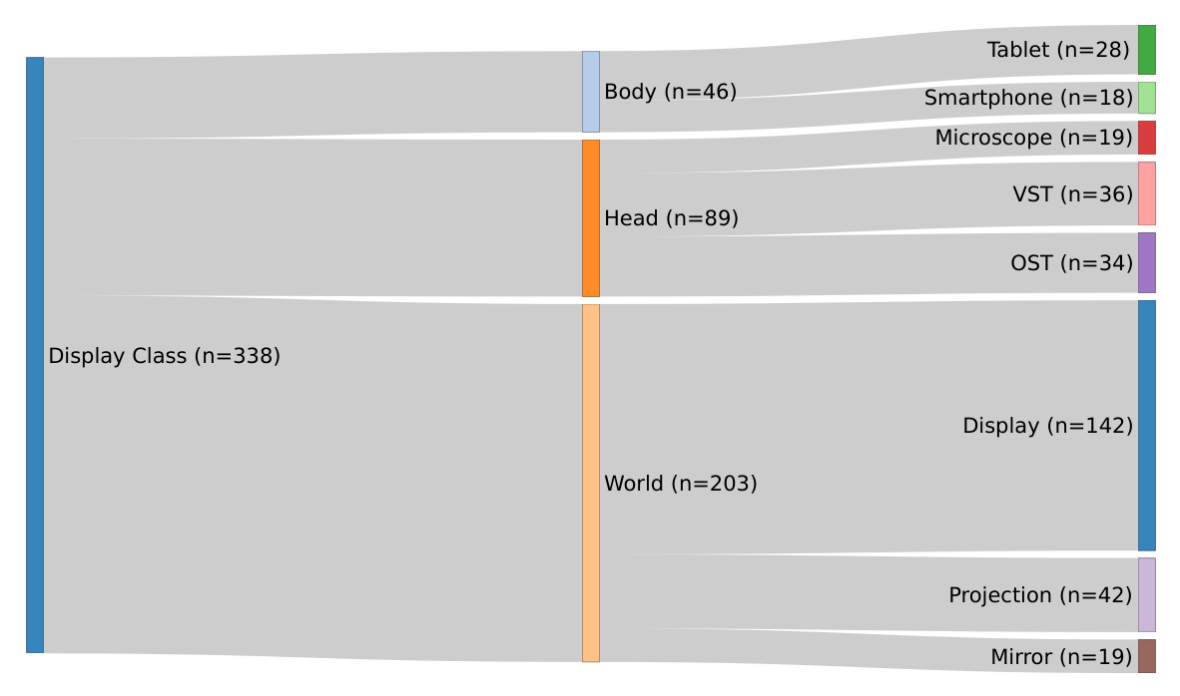
Categorization of display according to Schmalstieg et al [5] (N=338).

The results of the Display classification are shown in Figure 6. The Sankey plot shows that most of the displays are classified as “World,” and most of those have a common “Display.” For example, Kranzfelder [40] presented a system to add information from computerized tomography onto the images of gastrointestinal endoscopy. In the stream “Head,” one exemplary study by Carenzo et al [41] used Google Glass in a disaster medicine scenario as a tool to provide a triage algorithm in order to help assign triage codes to those injured in a disaster situation.

**Table 8:** Projection of technology readiness level distribution for all publications (N=338).

Technology readiness level	Number of publications
2	3
3	29
4	80
5	45
6	94
7	71
8	6
9	10

**Table 9:** Overview of the variables Mobile and Stationary Movement Tracking (N=338).

Movement tracking	Use of the tracking system		
	Yes	No	None
Mobile	247	63	28
Stationary	134	176	28

Within the stream “Body,” some of the applications used a “Tablet” to visualize 3D information from a medical imaging system. For example, a system used a tablet showing a previously virtually reconstructed tumor during a neurosurgical operation, to assist the surgeon while drilling [42]. Another example is an app for medical students to learn about gunshot wounds [43].

Of all analyzed (N=338) publications, most optical tracking mechanisms are accomplished via a marker (n=223), mostly through commonly known mechanisms such as color variation (ie, chessboard pattern). This pattern was used by Edgcumbe et al [44], who applied the pattern of triangulation in a laparoscopic scenario. Natural Markers were used in 73 publications and a mixture of natural and marker-based optical tracking was used in 3 publications; no optical tracking was used in 39 papers.

The results for the stationary and mobile tracking mechanisms are presented in Table 5.

To avoid doubling numbers, for any device using more than one tracking mechanism, the stationary device was chosen. This occurred mostly in the Head Location or Video-See-Through scenarios, since there are devices like the HTC Vive or the Oculus Rift that depend on tracking with infrared, which are found in a stationary device or involve smaller movements that often rely on gyroscopes.

Table 9 shows that 134 of the analyzed publications used a stationary tracking system, while 28 used no tracking system and 176 did not use a stationary system. It is possible to track movement in a mobile manner, and this maneuver can be applied in a medical field. Mobile Tracking was provided in 247 of the presented apps.

In some cases, there was no tracking of movement, like the app for rehabilitation proposed in the study by Chinthammit et al [45], who overlaid an image produced by a trainer onto the reality from a patient, so that the patient could mimic the movement.

### Classification by Hugues et al

**Figure 7:**
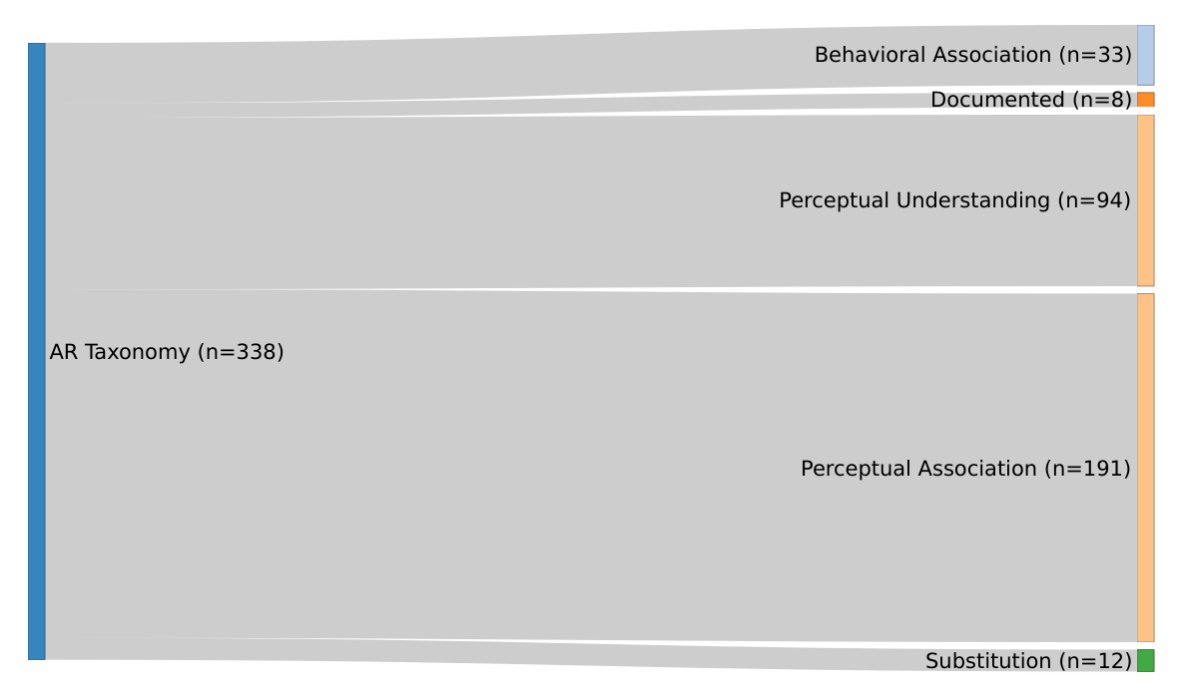
Categorization according to the taxonomy of Hugues et al [4] (N=338).

Figure 7 shows that of the 338 analyzed papers, in most cases (n=191, 56.5%), a “Perceptual Association” between the real object and the virtual object can be examined. For example, KleinJan et al [46] developed an app for the declipseSPECT, a device that can present preoperative data from digital imaging and communications in medicine files in 3D. This app can combine the AR aspect of the device with fluorescence imaging by adding a fluorescence layer into the scenario, making it easier for surgeons to track and extract sentinel nodes in surgeries.

In the second most classified functionality—“Perceptual Understanding”—there is a connection between the real and virtual environment, similar to the app presented by Marker et al [47]. They implemented a method to add information to a screen while it is being visualized in a magnetic resonance imaging system. This approach supports the surgeon to target the paravertebral space.

The functionalities “Behavioral Association” and “Substitution” are addressed in 35 of the analyzed papers. One example is the paper by Olivieri et al [48] about an app that monitors the user via electroencephalography and gives feedback based on the concentration of the participant while training in a surgical simulation. Eleven published apps show only additional information that is unrelated to the reality that surrounds the user. This can be seen, for example, in the study by Wilson et al [49], who developed an app to treat a tension pneumothorax by decompressing it. The device showed the steps necessary to fulfill this treatment, which did not depend on where the user was looking.

## Discussion

### Principal Findings

Evaluating the recent publications in the analysis timeframe, we detected an increasing trend in the number of papers on AR. This is most likely because the technology is developing, and affordable systems are partially available in daily life. In most cases, the first author was located in the United States, followed by Germany, Japan, and France.

Inspecting only the PubMed database and their MeSH annotations, the dominating terms were found to be Computer-Assisted Surgery, Three-Dimensional Imaging, Computed X-Ray Tomography, and Laparoscopy. All terms were connected to technology-intensive environments that make use of screens and advanced visualization technology. This finding was also observed in the analysis of the Medical Scope, as the results show that the main scope was Treatment, and within this area, primarily, the operating room.

With only three clinical trials registered in ClinicalTrials.gov, the scope for clinical trials was unexpected. One possible explanation for the low number of registered trials could be that it is not mandatory to register a medical invention prior to a clinical trial. Furthermore, most techniques were at a technology readiness level of 6 to 7; thus, they were mostly prototypes but needed additional completion and proving, a step that would lead to a clinical trial. We often observed that trials were conducted, but with a low number of subjects (N<9), or that the trials conducted were animal trials, which are not registered.

Only a very small subset of explored AR technologies featured a collaboration aspect. This could be due to the lack of widely available network coverage in sensitive environments or simply the size of AR technologies. Wearable technologies, in particular, are still too bulky to be used in a professional setting.

The maturity analysis of the research projects showed that the majority of publications were distributed in a rough bell-curve shape around level 6 and the use of technology was demonstrated in a relevant environment. This indicates that either early research is not published or basic research is no longer necessary, because AR technology is underway to production readiness.

From a medical perspective, the majority of papers were categorized under “Treatment.” This is due to the fact that the use of displays is already established in operating rooms, and this is a small step toward adding information to the images shown.

This finding is additionally supported by the next categorization; the technological perspective showed that the majority of publications were written under the stream “World,” and in these cases, the majority were in the class “Display.”

The training scope is dominated by surgery simulations. Since the technology is easy to apply in a training environment, this is an expected finding.

The second most important role after the presentation of the augmented world is the tracking of real-life objects that are used in the augmented space. Most of the tracking mechanisms used “marker-based” optical tracking, a technique that, in most cases, is more robust and accurate than “markerless” optical tracking. Only 39 publications did not use any kind of optical tracking, as it was not necessary for their apps. The majority of projects used a mobile tracking system. This can be explained by the broader range of movements provided by mobile tracking systems, which is essential in most cases, especially in surroundings like a hospital room.

Defining the different levels of augmentation based on the taxonomy by Hugues et al, majority of the apps were assigned to the class “Perceptual Association”; thus, most apps seek to make associations between the real and the virtual world, for example, the association between an organ seen in endoscopy and the visual image projected onto it. The second most observed definition was “Perceptual Understanding”; the virtual object draws a connection to what is seen and explains or shows certain objects in the real world.

### Strength and Limitations

The strength of manually analyzing the publications is that data could be deducted, which is not explicitly written, and were therefore not detectable by automated text mining. For example, some articles stated which device was used but did not disclose the tracking technology or which display was used explicitly. Since specifications for most devices are known, the basic tracking and display technologies were added to the result, as derived from context, for example, the publication by Shi et al [50] who presented an AR app in combination with a robot. The robot used fuzzy controlled mechanisms to help surgeons control a drill during a mandible plastic surgery; the surgeons can see additional information about the bone structure through AR glasses.

An additional strength of manual analysis is that whenever ambiguous terms were used, it could be compensated. If a system was introduced as AR but was, by the Milgram definition, a VR system, the paper was excluded. In contrast, publications were included even if they involved ambiguous use of the term “virtual,” as seen in the study by Lozano-Quilis [51], who proposed a “virtual rehabilitation” scenario, showing and explaining an AR environment.

One strength of this review, in contrast to that by Chen et al [2] who used only Scopus to retrieve published papers, is that this paper used two databases (PubMed and Scopus) to find the initial set of publications.

The number of analyzed publications is, in contrast to the study by Chen et al [2], a dataset limitation. Another limitation is that publications without an abstract and not containing AR in the title were not identified in our search. Because the publications were analyzed manually, there is a possibility of an individual bias of the analyzing author. To prevent this bias, the authors cross-checked 25% of the publications.

### Conclusions

This work provides a detailed view into 5 years of AR research in medicine. AR in medicine is an emerging technology that can benefit medical practitioners, health care professionals, and patients. The assessment of the technology readiness level shows that the AR technology is beyond the testing phase, and practical applications are becoming more common.

MeSH term analysis showed that the fields of Computer-Assisted Surgery, Three-Dimensional Imaging, and Computed X-Ray Tomography are the most explored. These fields already make use of advanced display technologies, and it is easy to integrate AR technologies into their workflow.

There was also a clear trend in technologies assisting actual treatment of patients in comparison to technologies in a training environment. In the treatment/training aspect, a more balanced distribution of publications was expected.

AR technology is an upcoming technology that will impact the treatment of patients in the future. With shrinking and more powerful hardware, the technology will be able to merge better into existing workflows and create opportunities for patients, doctors, and health care professionals.

The aim of this review was to offer a foundation to researchers, which was met by offering a categorized list. A certain scope of research can be searched from our list by using the interactive table in Multimedia Appendix 1, which can, for example, be filtered to fit a certain category or criteria.

In contrast to the study by Chen et al [2], the authors expanded the data source. Chen et al [2] only accessed the Scopus database, whereas we used PubMed and Scopus databases as a basis for analysis. Therefore, a more comprehensive dataset was created. Another advantage of manual analysis is the ability to analyze graphics and information that are not directly contained in the text. An analysis using text mining is limited to recognizing synonyms and structures and displaying them.

## Appendix

Multimedia Appendix 1 Interactive Excel table dataset.

Multimedia Appendix 2 Publications before first exclusion, including reviews.

Multimedia Appendix 3 Dataset of the complete reference list.

## Abbreviations

AR: augmented reality
AV: augmented virtuality
HMD: head-mounted display
MeSH: Medical Subject Headings
MR: mixed reality
SPECT: single-photon emission computed tomography
VR: virtual reality
